# Draft Genome Sequence of *Staphylococcus gallinarum* DSM 20610^T^, Originally Isolated from the Skin of a Chicken

**DOI:** 10.1128/genomeA.00580-15

**Published:** 2015-06-04

**Authors:** Ding Shi, Daiqiong Fang, Xinjun Hu, Ang Li, Longxian Lv, Jing Guo, Yanfei Chen, Wenrui Wu, Feifei Guo, Lanjuan Li

**Affiliations:** a State Key Laboratory for Diagnosis and Treatment of Infectious Disease, The First Affiliated Hospital, Zhejiang University, Hangzhou, People’s Republic of China; b Collaborative Innovation Center for Diagnosis and Treatment of Infectious Diseases, Hangzhou, People’s Republic of China

## Abstract

*Staphylococcus gallinarum* DSM 20610^T^ is a rare pathogen in humans. The increasing relevance of human health prompted us to determine the genomic sequence of *S. gallinarum*. The complete genome sequence of *S. gallinarum* includes a genome of 3,171,720 bp (33.02% G+C content) without any plasmids.

## GENOME ANNOUNCEMENT

*Staphylococcus gallinarum* DSM 20610^T^ was originally isolated from the skin of a chicken (1). *S. gallinarum* spp. are widespread in nature and have mainly been reported in poultry (2). They have also been isolated from saliva of healthy humans (3). The coagulase-negative staphylococcus (CoNS) *S. gallinarum* has been reported as an opportunistic human bacterial pathogen in some cases (4, 5). It has caused bacteremia in a patient with chronic hepatitis B virus infection, who presented with a low-grade fever, increased upper abdominal pain, nausea, and weakness (6). It can also cause traumatic endophthalmitis with resistance to caftazidime (7). *S. gallinarum* DSM 20610^T^ is a type strain. We determined the genomic sequence of *S. gallinarum* DSM 20610^T^ because of the increasing clinical relevance of this group.

DSM 20610^T^ was grown aerobically on a Columbia blood agar base at 37°C for 24 h. Genomic DNA was extracted using the DNeasy blood and tissue kit (Qiagen, Germany), as described earlier (8). Whole-genome sequencing was performed at the State Key Laboratory for Diagnosis and Treatment of Infectious at Zhejiang University using an Illumina HiSeq 2000 genomic sequencer. This is the first genome sequence of the species *S. gallinarum*. The shotgun library (412-bp insert size, with an Illumina adapter at both ends) was prepared according to the manufacturer’s protocols, with a 2 × 150 paired-end sequencing strategy. The reads were assembled using Velvet version 1.2.07 (9). Gene prediction was carried out by using Glimmer version 3.0 (10). The genome was annotated using HMMER version 3.0 (11). tRNAscan-SE version 1.21 (12) was used to find tRNA genes, whereas ribosomal RNAs were found by using RNAmmer version 1.2 (13). The KAAS server (14) was used to assign translated amino acids into KEGG orthology (15). Genes were aligned with the COG database (16).

The complete genome of *S. gallinarum* DSM 20610^T^ contained a 3,171,720-bp circular chromosome with no plasmid. The G+C content of the chromosome was 33.02% (272 scaffolds with an *N*_50_ of 3,483,00 bp). These scaffolds contain 2,988 coding sequences (CDSs), 57 tRNAs (excluding 3 pseudo-tRNAs), and 11 incomplete large subunit rRNA operons. A total of 794 protein-coding genes were assigned as putative function or hypothetical proteins. We categorized 2,412 genes into functional clusters of orthologous groups (including putative or hypothetical genes).

In summary, this new genome sequence will provide an improved basis to elucidate the molecular principles of this organism’s pathogenesis, and also for further investigation and application of the genotypic prediction of antimicrobial resistance of the coagulase-negative staphylococcus. Availability of the *S. gallinarum* DSM 20610^T^ genome could prompt the development of postgenomic tools for its rapid discrimination from *Staphylococcus* spp*.*

### **Nucleotide sequence accession number**s. 

This whole-genome shotgun project has been deposited at DDBJ/EMBL/GenBank under the accession no. JXCF00000000. The version described in this paper is version JXCF01000000.
